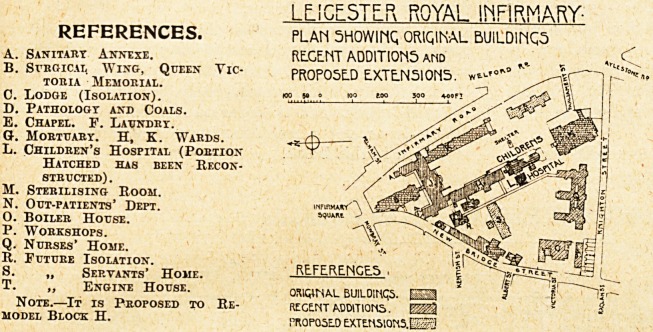# Leicester Royal Infirmary Children's Hospital

**Published:** 1916-11-04

**Authors:** 


### LEICESTER ROYAL INFIRMARY CHILDREN'S HOSPITAL.

We publish to-day plans of the ground, first, and second floors, with a block plan of the reconstructed children's hospital of the Leicester Royal Infirmary. The new work is indicated in solid block and the old work is hatched. It will be noticed that very little of the old building except the main walls has been retained, and that extensive additions have been made. Formerly the block consisted of two wards, with nurses' rooms on the top. This top storey has been entirely removed and a new ward constructed in its place, increasing the accommodation from forty-two to sixty-six cots.

The ground and first floor wards are for surgical cases, and the second-floor ward for medical cases. They are practically identical in plan, one end being semicircular. The long axis of the building is roughly N.W. and S.E.; at the south side of the wards spacious balconies are provided where the cots can be wheeled from the wards. The sides of these balconies are fitted with glazed folding screens which can be opened or closed on any particular side. Inside the wards the old wood-block floors have been removed and terrazzo substituted, and the old fireplaces in the side walls have been replaced by central double grates with descending flues taken under the floors and connected to a new stack of vertical flues in the side walls. The walls and ceilings are finished in white enamel and the new ward on the second floor has a decorative tile dado 5 ft. 6 in. high. The window spaces have been enlarged, the openings being cut down to the floor level and glazed doors fitted. A Berkefeld filter and a sink have been fixed in a central position in each ward for the use of the nurses and doctors.

The following accommodation, forming part of the old

Ground-floor Plan.

First and Second Floor Plan.
Everard, Son & Pick, F.R.I.B.A., Architects, Leicester.

structure in connection with these wards, has been retained. On the ground floor: kitchen, and one single-bed ward. On the first floor: kitchen, and two single-bed wards. A group of new rooms has been added on each floor, the accommodation being as follows:—Ground floor: nurses' sitting-room, linen-room, bathroom, and lavatory. First and second floors: nurses' sitting-room, clinical-room, linen-room, and bathroom. The position of the bathroom is rather unfortunate, but it is difficult to see how it could have been placed otherwise. It is always desirable in a children's ward to have direct access to the bathroom from the ward.

The old sanitary block remains, but has been provided with disconnecting lobbies or bridges at each floor level. The windows have been enlarged and the ventilation improved by the addition of hoppers over the casements. An electric lift has been fixed at the north-west end of the building to serve all floors, and an escape staircase has also been provided. On the south-west side on the ground floor an operation block has been built consisting of a theatre, wash-up room, nurses' room, anæsthetic room, and surgeon's room. Space for this block would appear to have been limited by the position of the covered way between two of the other blocks; otherwise a little more room might have been devoted to keeping the sterilisers and sinks outside the theatre. A direct access from the anæsthetic room to the theatre also would have been an improvement.

In carrying out extensive alterations in a building of this kind many difficulties in construction, etc., are bound to be met with, such as do not occur in an entirely new building, consequently it is not possible to obtain an ideal scheme in every respect. Taken as a whole the result is good, and the building is an excellent one for its purpose. Three fine wards have been obtained, with ample floor and cubic space for each patient. The work was carried out by Messrs. Everard, Son and Pick, of Leicester.

**REFERENCES.**

- A. Sanitary Annexe.

- B. Surgical Wing, Queen Victoria Memorial.

- C. Lodge (Isolation).

- D. Pathology and Coals.

- E. Chapel. F. Laundry.

- G. Mortuary. H, K. Wards.

- L. Children's Hospital (Portion Hatched has been Reconstructed).

- M. Sterilising Room.

- N. Out-patients' Dept.

- O. Boiler House.

- P. Workshops.

- Q. Nurses' Home.

- R. Future Isolation.

- S. " Servants' Home.

- T. " Engine House.

Note.—It is Proposed to Remodel Block H.

## Figures and Tables

**Figure f1:**
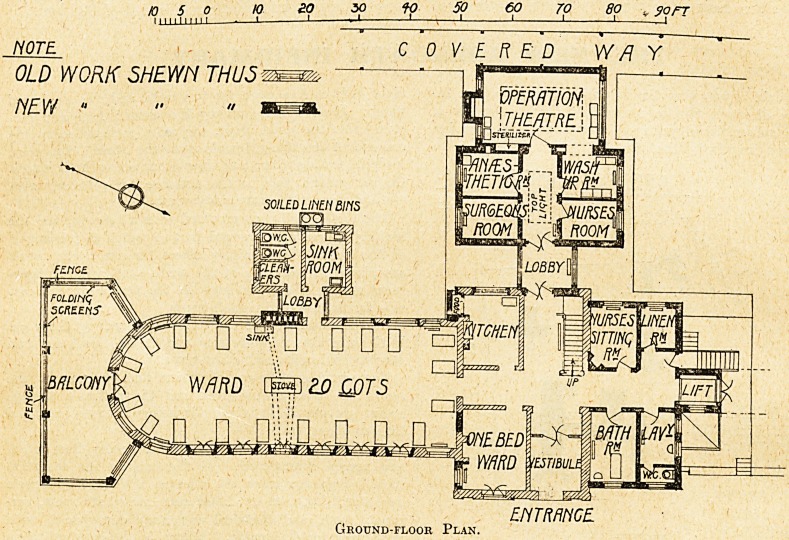


**Figure f2:**
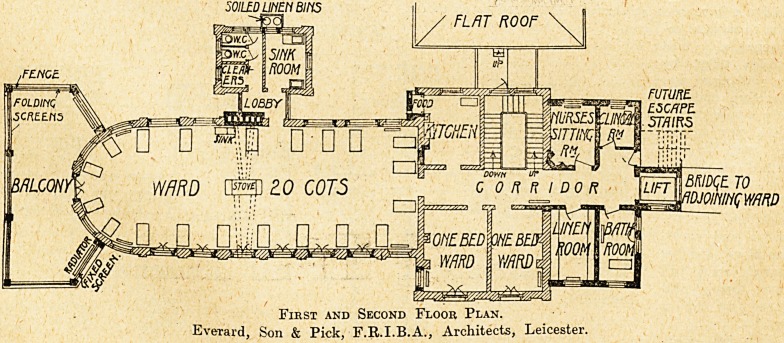


**Figure f3:**